# *In situ* study of defect migration kinetics in nanoporous Ag with enhanced radiation tolerance

**DOI:** 10.1038/srep03737

**Published:** 2014-01-17

**Authors:** C. Sun, D. Bufford, Y. Chen, M. A. Kirk, Y. Q. Wang, M. Li, H. Wang, S. A. Maloy, X. Zhang

**Affiliations:** 1 Department of Materials Science and Engineering, Texas A&M University, College Station, TX 77843; 2 Materials Science and Technology Division, Los Alamos National Laboratory, Los Alamos, NM, 87545; 3 Materials Science Division, Argonne National Laboratory, Argonne, IL 60439, USA; 4 Nuclear Engineering Division, Argonne National Laboratory, Argonne, IL 60439, USA; 5 Department of Electrical and Computer Engineering, Texas A&M University, College Station, TX 77843; 6 Department of Mechanical Engineering, Texas A&M University, College Station, TX 77843

## Abstract

Defect sinks, such as grain boundaries and phase boundaries, have been widely accepted to improve the irradiation resistance of metallic materials. However, free surface, an ideal defect sink, has received little attention in bulk materials as surface-to-volume ratio is typically low. Here by using *in situ* Kr ion irradiation technique in a transmission electron microscope, we show that nanoporous (NP) Ag has enhanced radiation tolerance. Besides direct evidence of free surface induced frequent removal of various types of defect clusters, we determined, for the first time, the global and instantaneous diffusivity of defect clusters in both coarse-grained (CG) and NP Ag. Opposite to conventional wisdom, both types of diffusivities are lower in NP Ag. Such a surprise is largely related to the reduced interaction energy between isolated defect clusters in NP Ag. Determination of kinetics of defect clusters is essential to understand and model their migration and clustering in irradiated materials.

The successful development of advanced nuclear reactors calls for the discovery of advanced materials that can endure unprecedented neutron irradiation damage to hundreds of displacements-per-atom (dpa)^1^,^2^,^3^. A high density of irradiation-induced defect clusters, including dislocation loops and networks, voids, bubbles and stacking fault tetrahedra (SFTs), can significantly degrade mechanical properties of materials^4^,^5^,^6^,^7^. Several types of defect sinks have been explored to achieve enhanced radiation tolerance, such as high-angle grain boundaries (GBs)^8^,^9^,^10^,^11^,^12^,^13^, immiscible interfaces in nanolayer composites^14^,^15^,^16^,^17^, twin boundaries^18^,^19^ and phase boundaries^20^,^21^. Metallic nanoporous (NP) materials with large surface-to-volume ratios have applications for energy storage, catalysts, filters and gas sensors^22^. Their mechanical, catalytic and optical properties have been widely investigated^23^,^24^,^25^. Lee et al.^26^ reported mechanical strength of NP Au and suggested that NP metals could be used as high strength, low density materials. Kucheyev et al.^27^ concluded that the pronounced time-dependent creep of NP silica at room temperature was attributed to the stress corrosion fracture of nanoscale ligaments. The impact of free surface on irradiation-induced damage in bulk materials has also been studied. In general a larger number of defect clusters (mostly vacancy loops) were observed near-surface compared to those in materials interior (inside bulk materials)^28^,^29^,^30^,^31^. Such disparity in defect distribution has been explained by preferential removal of mobile interstitials by free surfaces, leaving less mobile vacancy clusters behind (near surfaces)^29^. MD simulations also observed viscous flow of atoms to free surfaces^32^. Norris et al.^33^ reported void denuded zones near the free surface of Ni foil under high voltage electron beam irradiation. Bringa et al.^34^ predicted via MD simulations that, at a given dose rate, the Au foam would be resistant to radiation if the ligament diameter is within an optimum window. The ligament, if too small (several nm) would melt and break during radiation, and, if greater than 100 nm, would accumulate damage rapidly, similar to bulk materials. Using *ex situ* Ne ion irradiation, Fu et al.^35^ showed the formation of SFTs in porous Au is dose rate dependent.

Despite prior studies on surface sinks, several significant issues remain unaddressed. First, there is no *in situ* evidence to unambiguously reveal defect removal and accumulation mechanisms via surfaces in NP metals. Second, defect migration kinetics, or diffusivity of nanoscale defect clusters in porous as well as conventional metals remains largely unknown^36^,^37^,^38^. Matsukawa and Zinkle^36^ reported a maximum instantaneous diffusivity of ~3000 nm^2^/s of vacancy clusters (3 nm in diameter) in Au during 1D migration along a dislocation line at ~113 K. Arakawa et al.^37^ showed global diffusivity (considering dwell time) of isolated dislocation loops (6 nm in diameter) in Fe to be ~50 nm^2^/s at 575 K. Most modeling studies focused on migration kinetics of subnanometer defect clusters, and hence the adopted diffusivity value in the modeling is rather large, typically in the range of 1 × 10^3^–2 × 10^10^ nm^2^/s^34^,^39^,^40^. Limited information on diffusivity of nanoscale defect clusters leads to significant uncertainty on prediction of defect accumulation and microstructure evolution in irradiated materials.

Here we performed the first *in situ* Kr ion irradiation on CG and NP Ag and directly revealed superior radiation tolerance of NP Ag. *In situ* video captured the migration and removal of irradiation-induced various types of defect clusters by free surface in NP Ag. We also determined the significant difference in global and instantaneous diffusivity of defect clusters in CG and NP Ag under irradiation. Our findings provide unambiguous evidence for understanding the significance of free surface on defect removal and migration kinetics in irradiated metallic materials, and thus offer new insight on design of radiation tolerant advanced materials by tailoring nanoscale porosity.

## Results

### Superior radiation tolerance of NP Ag over CG Ag

**Figure 1:**
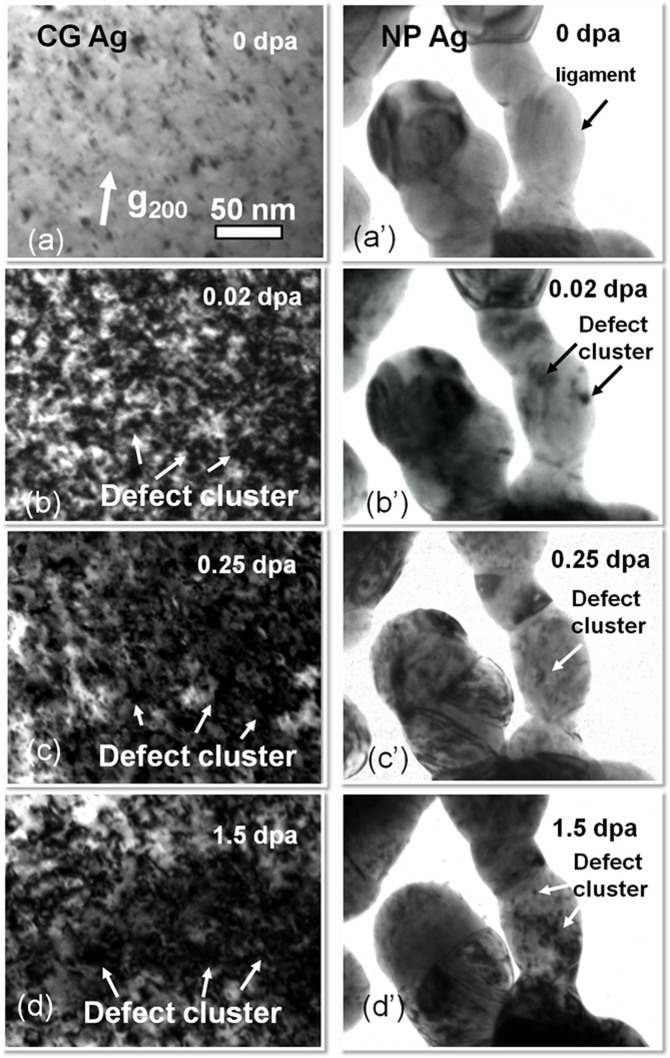
Drastic difference between microstructures of CG (a–d) and NP (a′–d′) Ag subjected to *in situ* Kr ion irradiation at room temperature. (a-a′) Annealed CG Ag films had a low density of preexisting defects, whereas NP Ag was nearly defect free. (b-b′) After irradiation to 0.02 dpa, CG Ag was swamped with a large number of defect clusters, whereas NP Ag remained intact (see Supplementary Video 2). (c-c′) By 0.25 dpa, there was a significant increase in both the size and density of defect clusters in CG Ag. In parallel few defect clusters were observed in the ligaments of NP Ag. (d-d′) By 1.5 dpa, the average defect cluster size in CG Ag appeared much greater than that in NP Ag.

**Figure 2:**
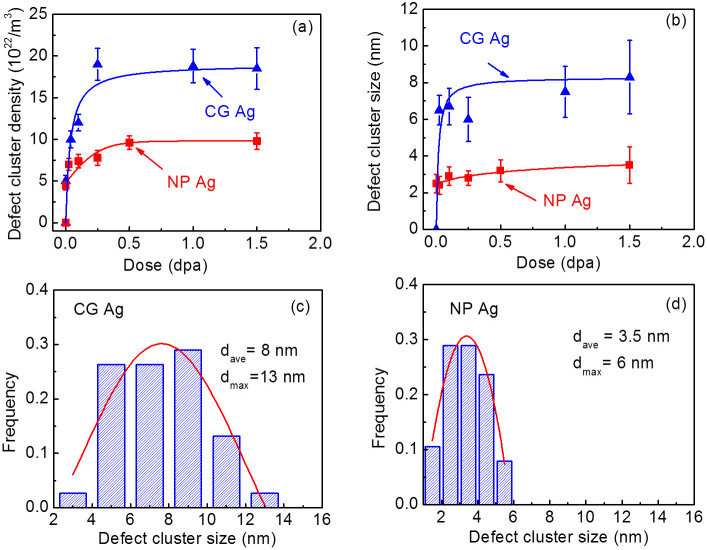
Statistics of density and size of defect clusters reveals superior radiation tolerance of NP Ag. (a) The density of defect clusters in NP Ag increased gradually with dose (fluence) to a saturation density of 1 × 10^23^/m^3^ at 0.5 dpa, whereas cluster density in CG Ag increased drastically and saturated by 0.25 dpa at twice higher value, 2 × 10^23^/m^3^. (b) At increasing dose, the average size of defect clusters in CG Ag increased monotonically to ~8 nm, whereas the defect cluster size in NP Ag remained small, ~3.5 nm by 0.5 dpa. (c–d) Statistical size distributions of defect clusters (by 1.5 dpa) show that CG Ag has both greater average and maximum defect size, ~8 and 13 nm respectively, comparing to 3.5 nm and 6 nm in irradiated NP Ag.

As-prepared NP Ag was transparent to the electron beam and had discrete islands which were mostly connected by ligaments (See supplementary Fig. S1a). Statistical study (supplementary Fig. S1b) shows the average ligament and island size is 40 and 150 nm, respectively. Selected area diffraction (SAD) pattern (in supplementary Fig. S1a) suggests the formation of polycrystalline NP Ag films with fine grains. Fig. 1 compares the microstructural evolution in NP and CG Ag under Kr ion irradiation at room temperature at an energy of 1 MeV. Some preexisting defect clusters (produced by annealing on rigid substrate) were observed in CG Ag before radiation (Fig. 1a). In comparison, NP Ag is basically free from obvious defect clusters prior to radiation (Fig. 1a′). *In situ* videos show that within the first 5–10 sec, defect density in CG Ag increased rapidly, whereas no detectable defect clusters formed in NP Ag (see supplementary Video 1 (0–0.014 dpa). By 0.02 dpa, the CG Ag was already swamped with a large number of defect clusters (Fig. 1b), whereas NP Ag remained intact (Fig. 1b′) (see Supplementary Video 2 (0.02–0.038 dpa)). Up to 0.25 dpa, there was a significant increase in both the size and density of defect clusters in CG Ag (Fig. 1c). In parallel few defect clusters were observed in the ligaments of NP Ag. By 1 dpa, *in situ* radiation (see supplementary Video 3) shows abundant defect migration activities in irradiated CG Ag, whereas only a handful of activities for defect clusters were captured in NP Ag. Finally by 1.5 dpa, the average defect cluster size in CG Ag appeared much greater than that in NP Ag (Fig. 1d and Fig. 1d′). Statistical studies show that defect cluster density in CG Ag (Fig. 2a) dramatically increased at low dose and reached saturation, ~2 × 10^23^ m^−3^ at ~0.25 dpa, while NP Ag approached a 50% lower saturation defect density, 1 × 10^23^ m^−3^ at 0.5 dpa. Meanwhile the average size of defect clusters in NP Ag shown in Fig. 2b reached a saturation value of ~3.5 nm, much lower than that in CG Ag, ~8 nm. Statistic studies in Fig. 2c–d show that after irradiation to 1.5 dpa, both the average and maximum sizes of defect clusters in NP Ag were much smaller than those in CG Ag.

For validation purposes, Kr ion irradiations at 800 keV at room temperature were performed *ex situ* on CG Ag TEM thin foils with nearly no preexisting defect clusters. SRIM simulations (Suppl. Fig. S2) show the radiation damage level in Ag within the depth of the first 100 nm is very similar between the two radiation conditions (*ex situ* 800 keV vs. *in situ* 1 MeV Kr ions). The microstructural evolution with irradiation doses up to 1 dpa (as shown in suppl. Fig. S3) suggests *ex situ* irradiated CG Ag exhibited nearly identical defect density compared to the *in situ* irradiated CG Ag at the same dose level. Bright field and weak beam dark field (WBDF) images (suppl. Fig. S4) of irradiated CG Ag (at 1 dpa) show that a majority of defect clusters were dislocation loops and SFTs.

### *In situ* evidence of defect cluster removal in NP Ag

**Figure 3:**
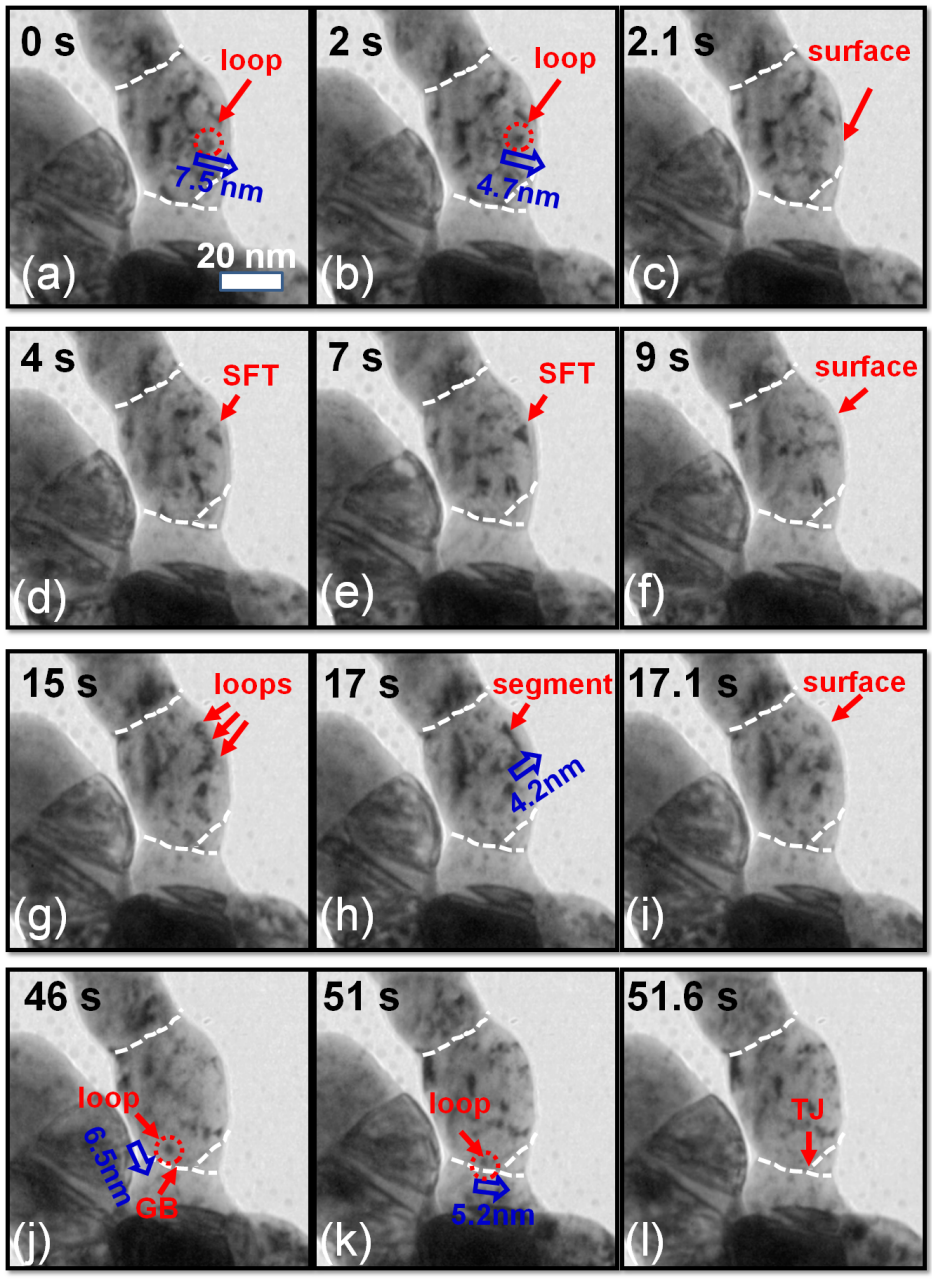
*In situ* video snap shots revealing several representative defect capture events by free surface or triple junctions (TJs) over 1.18–1.27 dpa. (See supplementary video 4 for details). (a–c) Evidence of rapid absorption of individual dislocation loops by free surface. A loop, ~7.5 nm away from free surface, migrated leftwards in 2 s, and was immediately removed by the free surface by 2.1 s. (d–f) A stacking fault tetrahedron (SFT) was gradually removed by the free surface from 7 to 9 s. (g–i) Formation of a dislocation segment and its rapid absorption by free surface. At 15 s, several individual dislocation loops were in contact with one another. The loops then combined to form a dislocation segment in 2 s at ~4.2 nm from free surface. Within 0.1 s, the dislocation segment was completely absorbed by the free surface. (j–l) Absorption of a dislocation loop by a TJ in NP Ag. At 46 s, a dislocation loop was generated near a grain boundary (GB). After 5 s, the loop was rapidly attracted towards the GB within 0.1 s, and the loop migrated along the GB for 0.6 s before being captured by the TJ.

We now present several typical examples in NP Ag where defect clusters were absorbed by defect sinks under irradiation (see supplementary Video 4 for details). First we report the removal of individual dislocation loops by free surface (Fig. 3a–c). A radiation induced dislocation loop (~4 nm in diameter) was located at 7.5 nm from free surface (Fig. 3a). By 2 s, the loop migrated 1.8 nm towards the free surface (Fig. 3b), and within 0.1 s, the loop was immediately absorbed by the free surface (Fig. 3c). Second, we examine the destruction of an SFT by a free surface (Fig. 3d–f). A large SFT, ~5 nm in edge length, was observed at 4 s, as shown in Fig. 3d close to free surface. The SFT remained stable till 7 s (Fig. 3e). However by 9 s, the SFT was gradually removed by the free surface (Fig. 3f). Third, *in situ* video snap shots (Fig. 3g–i) captured formation and rapid absorption of a dislocation segment. Several individual dislocation loops observed in ligament (Fig. 3g) at 15 s combined to form a dislocation segment, which was ~4.2 nm from free surface (Fig. 3h). Within merely 0.1 second, the dislocation segment was instantly absorbed by the free surface (Fig. 3i). Finally we look into the absorption of a discrete dislocation loop by triple junctions (TJs) in NP Ag (Fig. 3j–l). A dislocation loop outlined by a dash circle formed near a GB at 46 s (Fig. 3j). The dislocation loop rapidly migrated towards the GB (within 0.1 s) and then diffused along the GB towards a TJ for 0.6 s (Fig. 3k), and eventually was fully absorbed by the TJ (Fig. 3l).

### Drastic difference in defect migration kinetics between CG and NP Ag

**Figure 4:**
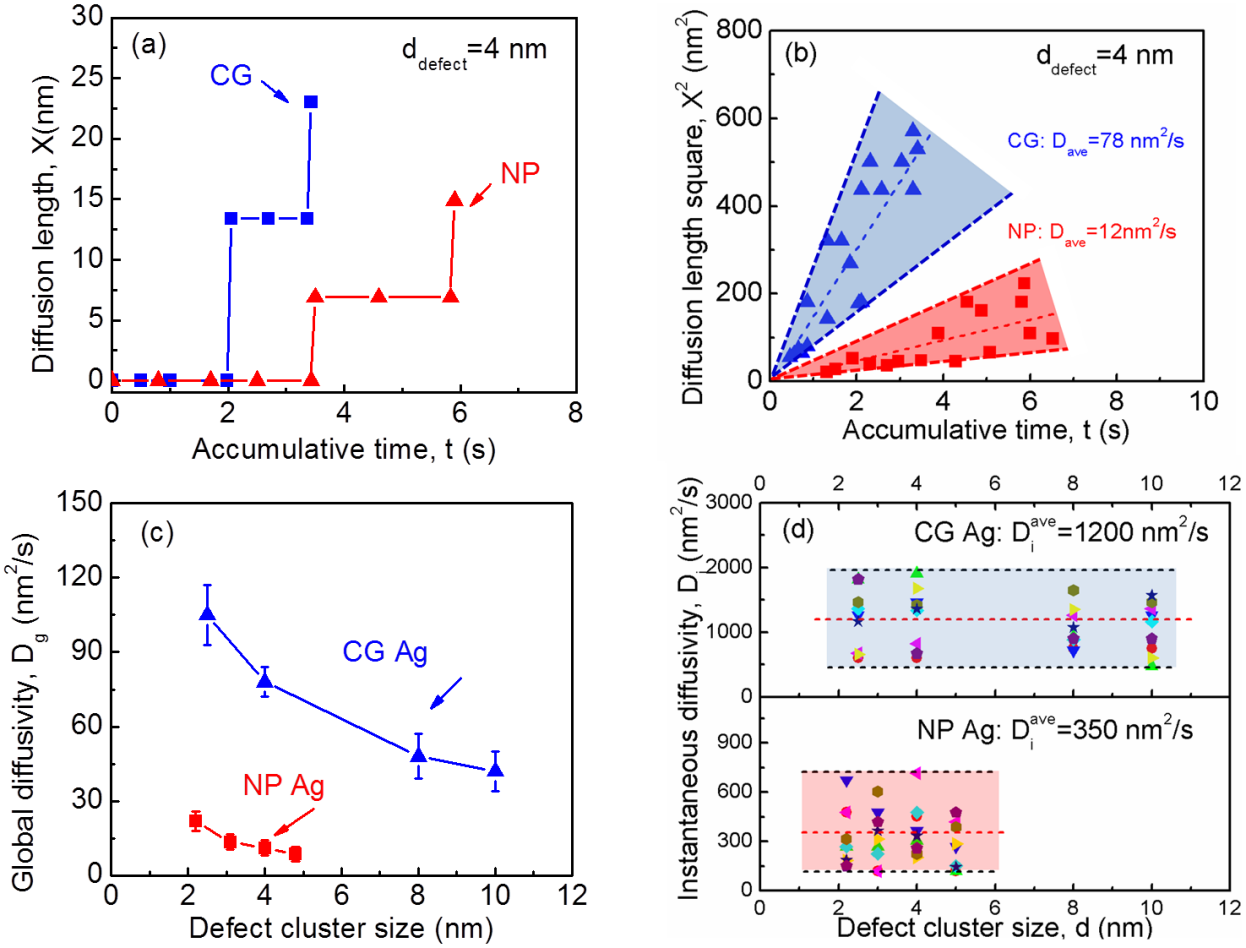
Determination of global and instantaneous diffusivity in Kr ion irradiated CG and NP Ag at a dose of ~1 dpa. (a) Representative plots of diffusion length (X) versus accumulative time for individual defect clusters with a diameter of 4 nm in CG and NP Ag. Defect clusters migrate in a similar “stick-jump” jerky pattern. (b) Plots of diffusion length square (X^2^) vs. accumulative time for a large number of defect clusters with a similar average size of 4 nm. The average global diffusivity was estimated to be ~78 and 12 nm^2^/s in CG and NP Ag, respectively. (c) The global diffusivity (D_g_) in both CG and NP Ag reduces monotonically with increasing defect cluster size. For nearly identical defect cluster size, the value of D_g_ of NP Ag is consistently lower than its CG counterpart. (d) However instantaneous diffusivity (D_i_) for both systems has no defect size dependence. The average value of D_i_ in CG and NP Ag was estimated to be 1200 and 350 nm^2^/s, respectively.

Besides vivid examination of various types of defect removal mechanisms, the *in situ* radiation study also provides abundant information to investigate defect migration kinetics in irradiated Ag. In particular we were able to determine global and instantaneous diffusivity of defect clusters. The global diffusivity (D_g_) is the diffusivity averaged over a long period of time (including migration and dwell time) for numerous defect clusters, whereas instantaneous diffusivity (D_i_) is measured only during the migration process. Fig. 4a shows typical examples of measured migration distance (diffusion length X) of individual defect clusters (4 nm in diameter) in both CG and NP Ag. In order to determine the global diffusivity of defect clusters, the migration of a large of number of defect clusters was studied statistically. Fig. 4b shows diffusion length square (X^2^) vs. accumulative time for numerous defect clusters with similar size (4 nm in diameter) for both CG and NP Ag. Assuming one dimensional diffusion, the diffusivity of defect clusters (D) can be estimated by *D* = *X*^2^/2*t*, where t is the diffusion time. Although there is error on the X^2^ measurement for both CG and NP Ag, a linear fit of these data shows that D_g_ of defect clusters (d_defect_ = 4 nm) in CG Ag is 78 nm^2^/s, much greater than that in NP Ag, ~12 nm^2^/s. Fig. 4c compares the size dependent variation of D_g_ for CG and NP Ag. Basically D_g_ reduced rapidly from 105 to 45 nm^2^/s with increasing size of defect clusters in CG Ag. A similar trend is observed in NP Ag. However, for the same dimension of defect clusters, D_g_ in NP Ag is consistently much lower than that in CG Ag. Similar techniques were applied to determine D_i_ as shown in Fig. 4d. Basically there is no clear size dependent variation of D_i_ for CG and NP Ag. Nonetheless the average value of D_i_ in NP Ag, ~350 nm^2^/s, is much less than that in CG Ag, ~1200 nm^2^/s. The value of D_i_ in both CG and NP Ag is at least an order of magnitude greater than their respective D_g_.

## Discussion

NP Ag has excellent radiation resistance as it contains enormous free surface that removes point defects and defect clusters and consequently significantly reduces the density and size of defect clusters in NP Ag compared with CG Ag. The absorption of point defects by free surface appears to result in defect concentration gradient and several nm wide surface-affected-zones (SAZs). The continuous flux of point defects towards free surfaces leads to drainage of overall point defect concentration in interior of NP Ag. Thus the formation and growth of defect clusters internally are significantly retarded.

Mechanisms for removal of SFTs, individual dislocation loop and dislocation segment by free surface might be different. (1) SFTs are highly stable defects in irradiated fcc metals with low stacking fault energy, and their removal typically requires high temperature annealing or mobile dislocations. SFTs within SAZs in NP Ag could be removed via numerous mechanisms. One of them could be accelerated migration of mobile SIAs in SAZ. These SIAs could interact with SFTs and lead to their collapse^18^. Another mechanism could be the direct interactions of apex of an SFT with free surface followed by dismantle of SFTs. Both mechanisms could lead to gradual removal of SFTs as observed during *in situ* irradiation. (2) The image force on discrete dislocation loops adjacent to free surface provides driving force for its migration towards free surface^41^. The image force arises from a virtual dislocation required to satisfy the free surface criteria (or balance the force induced by a real dislocation near free surface). By calculating image force on an edge dislocation without losing general applicability (see supplementary information for details of calculation), we estimate the vacancy formation energy near SAZ is ~0.04 eV. (3) In general there is insufficient defect supersaturation to support homogeneous cluster nucleation and growth next to planar defect sinks, such as free surfaces. Rapid exit of interstitials and vacancies in certain cases towards free surface leads to SAZ, where defect cluster density is negligible, consistent with previous study on void denuded zones close to surface of Ni foils^33^. Correspondingly, the density of defect clusters in NP Ag saturated at greater dose, ~0.5 dpa, compared to that in CG Ag, ~0.25 dpa. (4) Several individual dislocation loops in SAZ form a segment instead of being absorbed separately by free surface. The dislocation loops may have mutually attractive interaction forces (to be discussed in the next section), which balance with the image force from free surface. The combination of discrete dislocation loops to a single segment might eliminate such mutually attractive forces, and consequently the segment is rapidly absorbed by free surface.

**Figure 5:**
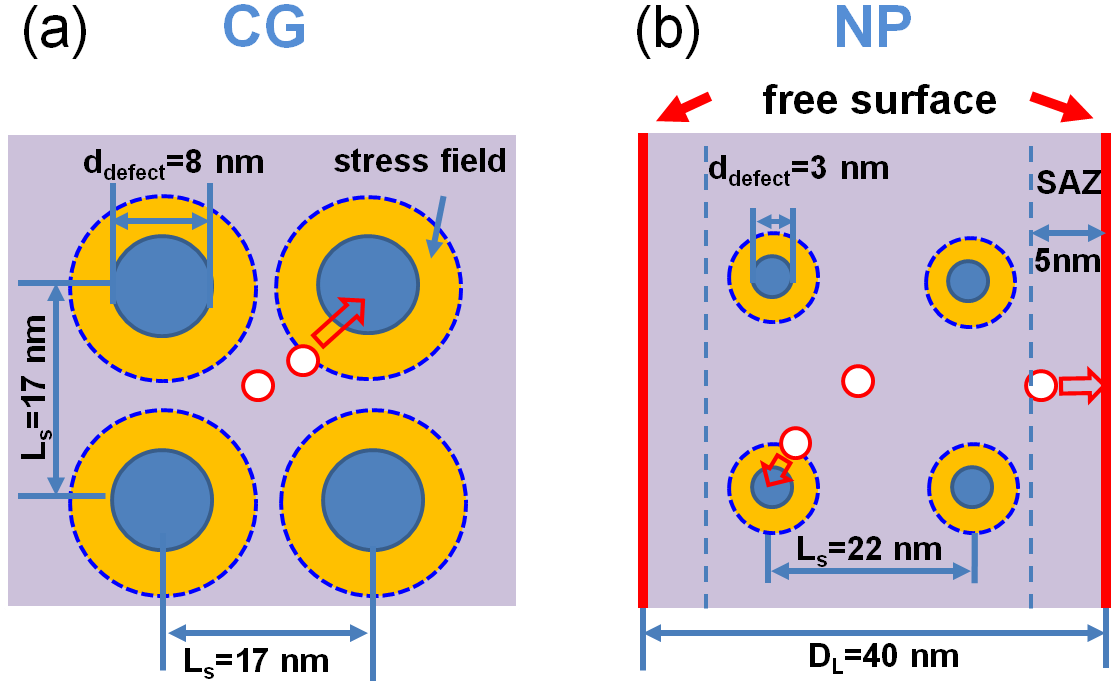
Schematic illustration on migration of defect clusters in CG and NP Ag. (a) In CG Ag, the average loop size and separation distance (L_s_) are 8 and 17 nm. The yellow zone outlines the region in which interactive stress field between the newly generated small defect clusters and preexisting large defect clusters is significant. As the stress field of these closely spaced large defect clusters tends to overlap, there is a greater probability to capture fresh radiation induced small defect clusters (indicated by open circles). (b) In NP Ag with ligament size (D_L_) of 40 nm, L_s_ among defect clusters (3 nm in diameter) is ~22 nm due to the lower defect cluster density. The width of the surface-affected-zones (SAZs) is ~5 nm. Radiation induced fresh small defect clusters can be removed by free surface (if they are located within SAZs). Compared with CG Ag, the preexisting defect clusters are much smaller, and have rather limited stress field (indicated by a narrower yellow zone). Furthermore these defect clusters are also more isolated, and consequently the possibility of capturing fresh radiation induced loop by preexisting loop is much less than in CG Ag.

Through *in situ* radiation experiments we determined D_i_ to be 350–1200 nm^2^/s in CG and NP Ag, orders of magnitude lower than values typically used in simulations^34^,^39^,^40^. Our study (Supplementary Video 1–3 and Fig. 4c) shows the expected trend of decreasing D_g_ with increasing defect size, while it is surprising to realize that defect clusters in NP Ag have actually lower D_g_ than in CG Ag. To understand this unexpected phenomenon, we speculate the interaction between coplanar dislocation loops in NP and CG Ag as follows. The interaction energy between defect clusters in CG Ag (d_defect_ = 8 nm, defect density of 2 × 10^23^ m^−3^) is calculated to be 0.85 eV (see supplementary Fig. S5 and calculation method), comparing to 0.06 eV in NP Ag (d_defect_ = 3 nm, defect density of 1 × 10^23^ m^−3^). The significantly reduced attractive force between defect clusters in NP Ag results in diminished possibility of mutual interaction and thus retards the global diffusivity of defect clusters. Fig. 5 illustrates schematically the interaction of defect clusters in irradiated CG and NP Ag. In CG Ag, larger dislocation loops generate stronger stress field (as shown in supplementary Fig. S5). Furthermore there is smaller spacing (L_s_ = 17 nm) between these large defect clusters as defect clusters density is very high. Consequently newly nucleated small defect clusters are prone to be trapped by these existing large defect clusters. In NP Ag, however, preexisting defect clusters are much smaller (3 nm) and more isolated from one another (separation distance L_s_ = 22 nm). Correspondingly the stress field of these smaller dislocation loops is less likely to allure newly generated tiny dislocation loops residing in more open spaces. Thus both diffusivities and the growth rate of defect clusters are significantly reduced. Of course as discussed earlier free surfaces in irradiated NP Ag play an important role on both counts as they not only absorb point defects but also remove defect clusters near SAZs, and thus limiting the density of defect clusters internally.

Foregoing discussions may also shed light to the phenomenon of lower D_i_ in irradiated NP Ag than CG Ag. It should be noticed that we might have underestimated the value of D_i_ to some extent limited by the speed of CCD camera (15 frame/s). The lack of size dependence for D_i_ in both CG and NP Ag is a surprising phenomenon, which may be elucidated further via modeling.

In summary, *in situ* Kr ion irradiation experiments allow us to explore the influence of free surface on the defect formation and defect migration kinetics in NP Ag. Comparing with CG Ag, NP Ag clearly exhibits significantly enhanced radiation resistance as evidenced by lower defect density and smaller defect size. *In situ* study provides direct evidence that free surface in NP Ag can effectively inhibit the formation of dislocation loops, dislocation segments and SFTs. We also determined global and instantaneous diffusivity of defect clusters in both systems. The observed much lower diffusivity of defect clusters in irradiated NP Ag than that in CG Ag, though counterintuitive at its first glance, is in fact consistent with enhanced radiation tolerance of NP Ag. Experimental determination of defect migration kinetics via *in situ* radiation provides crucial information to understand defect accumulation in irradiated metallic materials and could drastically improve the reliability of a variety of modeling studies on radiation damage.

## Methods

### Specimen preparation

Coarse-grained (CG) Ag films, ~2 μm in thickness, were prepared through magnetron sputtering by using 99.99% purity Ag target and oxidized Si substrates, and subsequent annealing. Prior to deposition, the chamber was evacuated to a base pressure of ~5 × 10^−8^ torr or better. Ultra high purity (99.99%) Ar gas pressure during sputtering was ~3.6 × 10^−3^ torr. NP Ag was produced by dealloying Ag_23_Cu_77_ (atomic%) films^42^, which were prepared by co-sputtering from pure Ag and Cu (99.99%) targets to a thickness of 200 nm. The films were etched with an 85% aqueous solution of phosphoric acid for approximately 48 hours to completely remove Cu. NP Ag was lifted directly onto carbon-coated grids for transmission electron microscopy (TEM) studies. TEM specimens were prepared at room temperature. TEM specimens were thinned significantly to minimize the time for Ar ion milling, which was performed at 3.7 keV at an incident angle of 5°. Low energy (2 keV) ion polishing was applied to remove ion milling induced damage in Ag.

### *In situ* and *ex situ* Kr ion irradiation

*In situ* Kr ion irradiation at 1 MeV was performed for CG and NP Ag at room temperature in the Intermediate Voltage Electron Microscope (IVEM) at Argonne National Laboratory, where an ion accelerator was attached to a HITACHI H-9000NAR microscope. The microscope was operated at 200 kV and kept on during radiation in order to record the microstructural evolution. The average dose rate was about 1.8 × 10^−3^ dpa/s. A CCD camera was used to capture microstructural evolution during radiation at 15 frame/second. SRIM^43^ simulation was used to estimate the displacement damage profile and Kr ion distribution. To check reproducibility and validate *in situ* radiation studies, *ex situ* Kr ion irradiation was performed at 800 keV at Ion Beam Materials Laboratory at Los Alamos National Laboratory. SRIM simulations (see supplementary Fig. S2) show that most of the Kr ions at 1 MeV and 800 keV will penetrate through the first 100 nm (the thickness of TEM foil is ~100 nm), whereas displacement damage will be contained primarily in the TEM foil; and the magnitude of depth dependent dose profile applied at both energy was controlled to be similar to each other.

## Author Contributions

C.S. and D.B. prepared specimens for *in situ* Kr ion irradiation; C.S. and Y.C. performed the *in situ* Kr ion irradiation experiments with assistance from M.K. and M.L. at Argonne National Laboratory. Y.Q.W. and C.S. performed *ex situ* Kr ion irradiation experiments at Los Alamos National Laboratory. X.Z., H.W. and S.A.M. assisted with interpreting the data. X.Z. developed the concept and directed the project. All authors discussed the results and commented on the manuscript.

## Supplementary Material

Supplementary Information Supplementary information

Supplementary Information Supplementary Video 1.

Supplementary Information Supplementary Video 2.

Supplementary Information Supplementary Video 3.

Supplementary Information Supplementary Video 4.
