# Supplementary material for: In situ study of defect migration kinetics in nanoporous Ag with enhanced radiation tolerance
Source: Sci Rep. 2014 Jan 17;4:3737. doi: 10.1038/srep03737 (PMC3894537; doi:10.1038/srep03737)
Supplement: Supplementary Information [file srep03737-s1.pdf]

In situ study of defect migration kinetics in nanoporous Ag with superior radiation tolerance

C. Sun[1,2], D. Bufford[1], Y. Chen[1], M. A. Kirk[3], Y. Q. Wang[2], M. Li[4], H. Wang[1, 5], S. A. Maloy[2], and X. Zhang[1, 6 *]

[1]Department of Materials Science and Engineering, Texas A&M University, College Station, TX 77843

[2] Materials Science and Technology Division, Los Alamos National Laboratory, Los Alamos, NM, 87545

[3] Materials Science Division, Argonne National Laboratory, Argonne, IL 60439, USA

[4] Nuclear Engineering Division, Argonne National Laboratory, Argonne, IL 60439, USA

[5]Department of Electrical and Computer Engineering, Texas A&M University, College Station, TX 77843

[6] Department of Mechanical Engineering, Texas A&M University, College Station, TX 77843

*Corresponding author: X. Zhang, zhangx@tamu.edu, Tel: (979) 845-2143

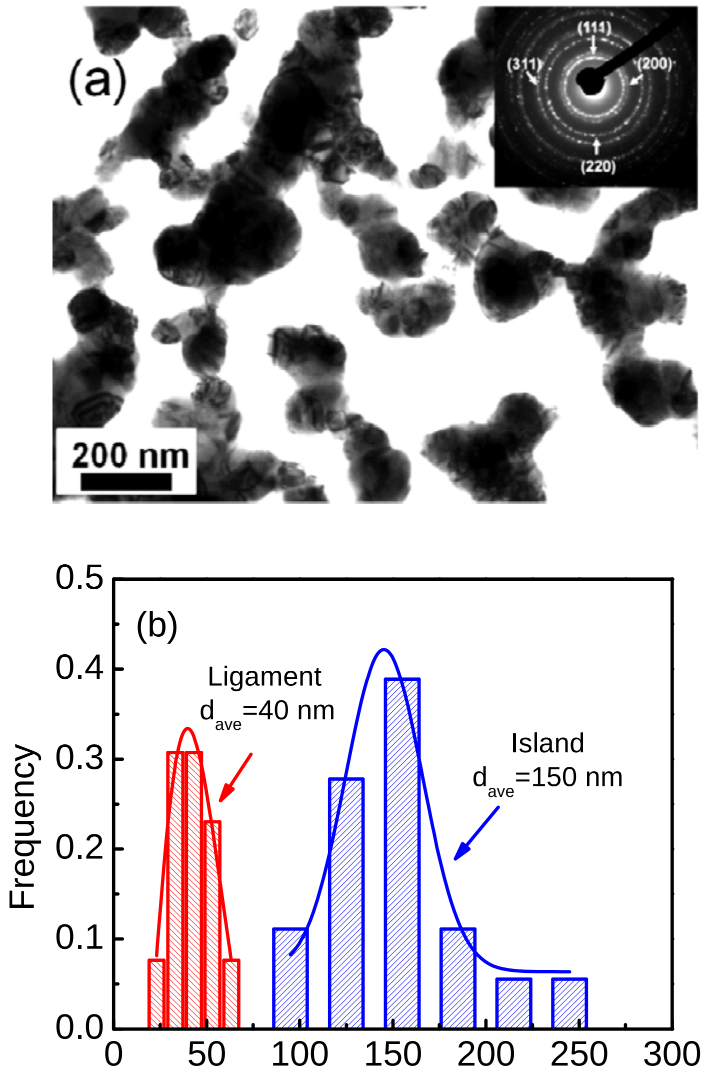

Supplementary Fig.S1. Microstructure of as-received nanoporous (NP) Ag films. (a) Bright field (BF) TEM image of NP Ag illustrating isolated and interconnected nanoscale islands. The inserted selected area diffraction (SAD) pattern shows nanocrystalline nature of NP Ag. (b) Statistical study on the size distribution of ligaments and islands in NP Ag. The average ligament and island size was 40 and 150 nm, respectively.

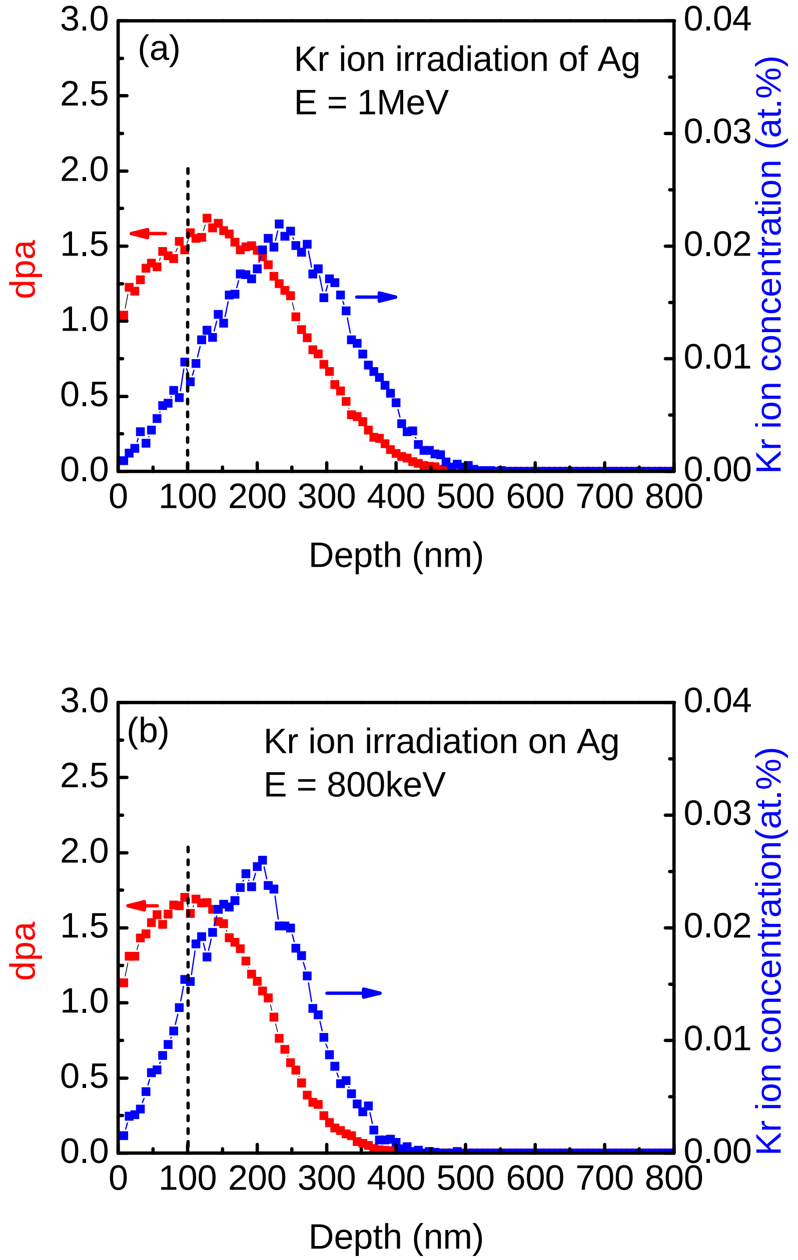

Supplementary Fig. S2. Depth profiles of radiation damage (dpa) and Kr ion concentration in Ag obtained from SRIM simulation by using Kinchin-Pease method at two incident energy, 1 MeV and 800 keV. (a) The energy of Kr ions is 1 MeV with a fluence of $3\times 10^{14}/cm^2$. At 100 nm from surface, the Kr ion concentration is ~ 0.01%. (b) The energy of Kr ions is 800keV with a fluence of $3\times 10^{14}/cm^2$. At 100 nm from surface, the Kr ion concentration is ~ 0.015%.

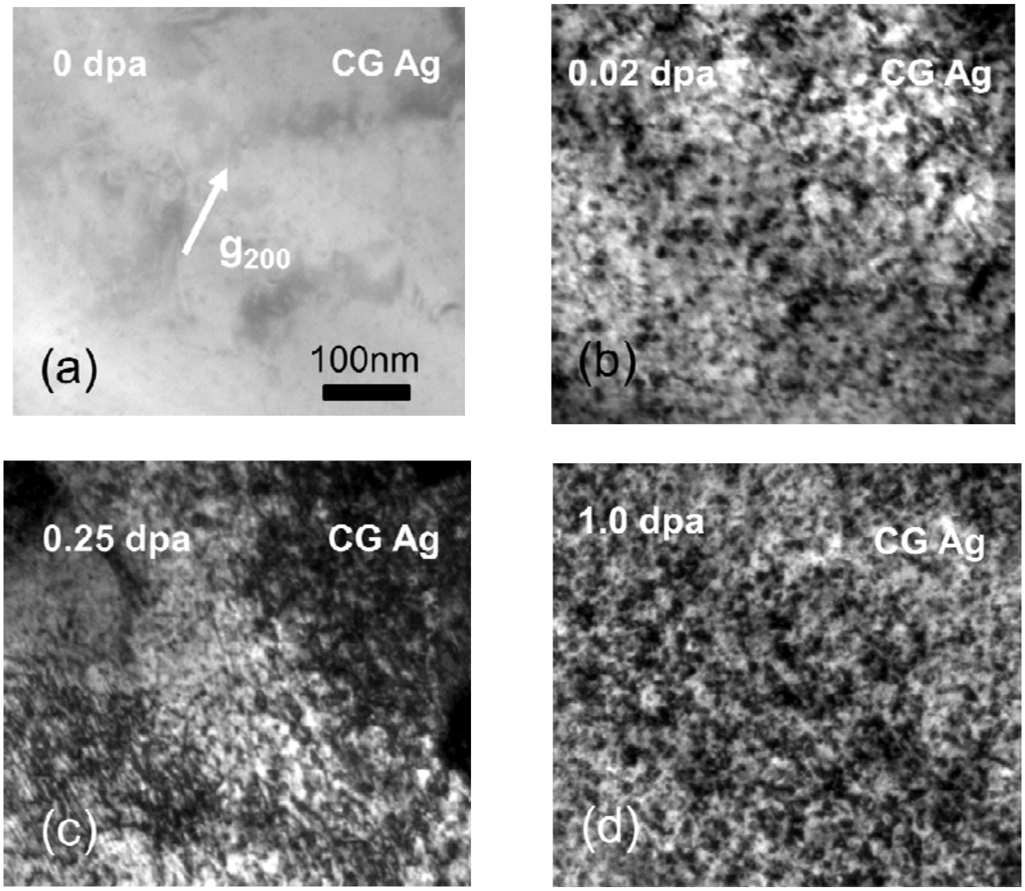

Supplementary Fig. S3. Microstructure evolution of CG Ag subjected to *ex situ* Kr ion irradiation at room temperature at an ion energy of 800 keV to different dose levels. (a) Annealed CG Ag films had very little preexisting defect clusters. After irradiation to 0.02 dpa, A large number of defect clusters were generated in CG Ag, which is similar to the microstructure of *in situ* irradiated CG Ag at an incidence energy of 1MeV. (c) By 0.25 dpa, there was a significant increase in both the size and density of defect clusters in CG Ag. (d) Up to 1.0 dpa, the average defect cluster size and density in CG Ag appeared to saturate to values nearly identical to those obtained from *in situ* (at 1 MeV) Kr ion irradiation.

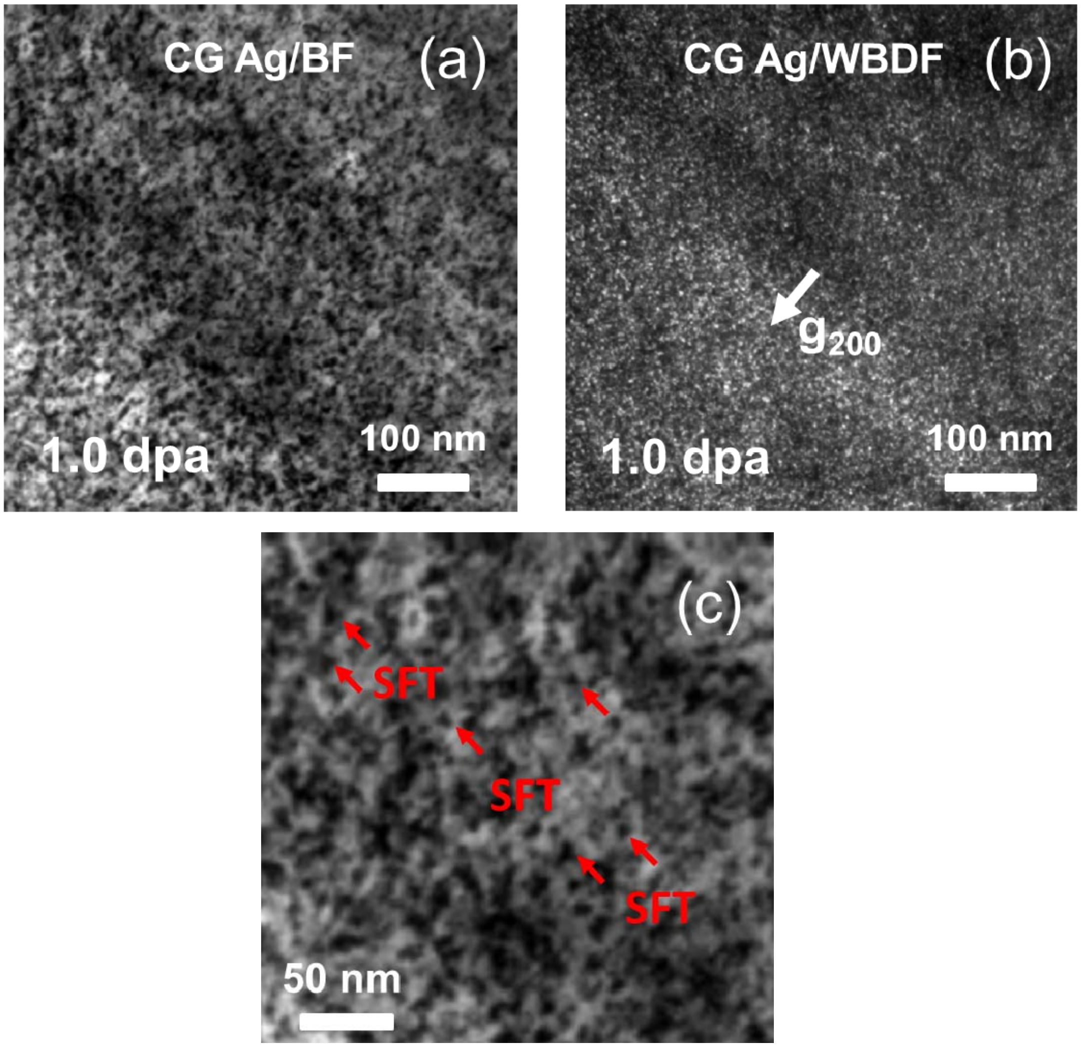

Supplementary Fig. S4. Microstructure of irradiated CG Ag after Kr ion irradiation at room temperature with ion energy of 800 keV to a dose of 1.0 dpa. (a) Bright filed (BF) TEM image of irradiated CG Ag with diffraction vector g = [200]. (b) Weak beam dark filed (WBDF) image of irradiated CG Ag with g/3g imaging condition. A large number of Frank loops was observed. (c) Stacking fault tetrahedra (SFTs) (indicated by red arrows) and dislocation loops were observed in irradiated CG Ag film.

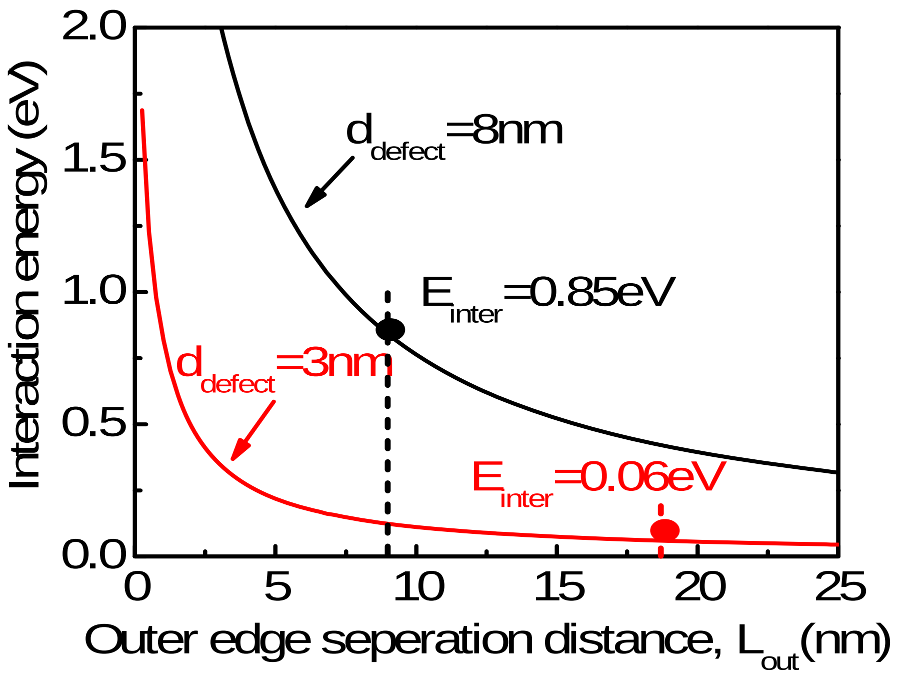

Supplementary Fig. S5. Evolution of interaction energy (E_{inter}) between two dislocation loops as a function of their outer edge separation distance (L_{out}) in CG and NP Ag. The interaction energy decreases rapidly with increasing L_{out}. At the same separation distance, E_{inter} between large loops (8 nm) in CG Ag is much greater than that between small loops (3 nm) in NP Ag. At the average L_{out} of 9 nm in CG Ag, E_{inter} of large loops is 0.85 eV, much greater than 0.06 eV in NP Ag at L_{out} of 19 nm.

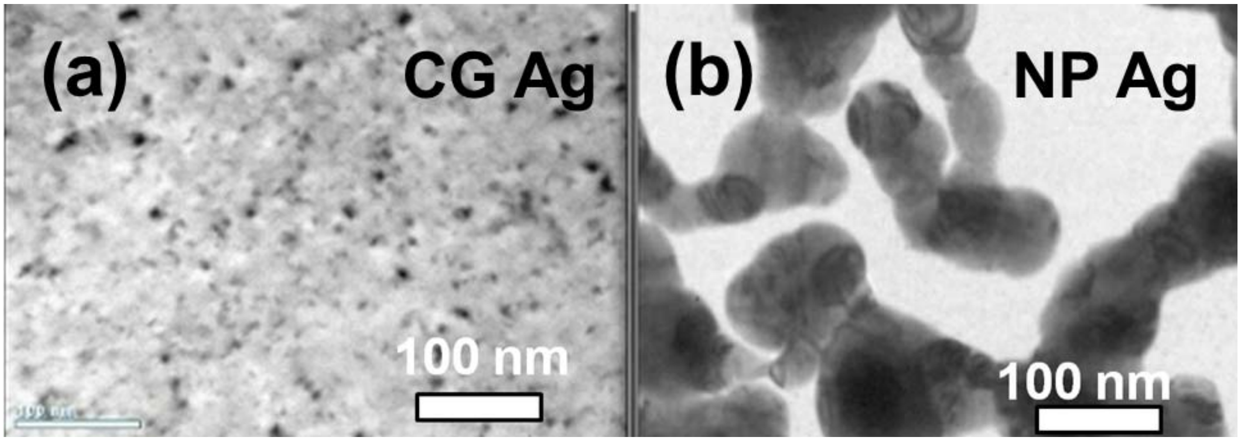

Supplementary Video 1. Comparison of irradiation response of CG and NP Ag subjected to Kr ion irradiation at room temperature from 0-0.014 dpa at energy of 1MeV. (a) TEM image of CG Ag. The video showed the explosive increase in defect density in CG Ag. (b) TEM image of NP Ag. The video shows the microstructure of NP Ag was barely changed.

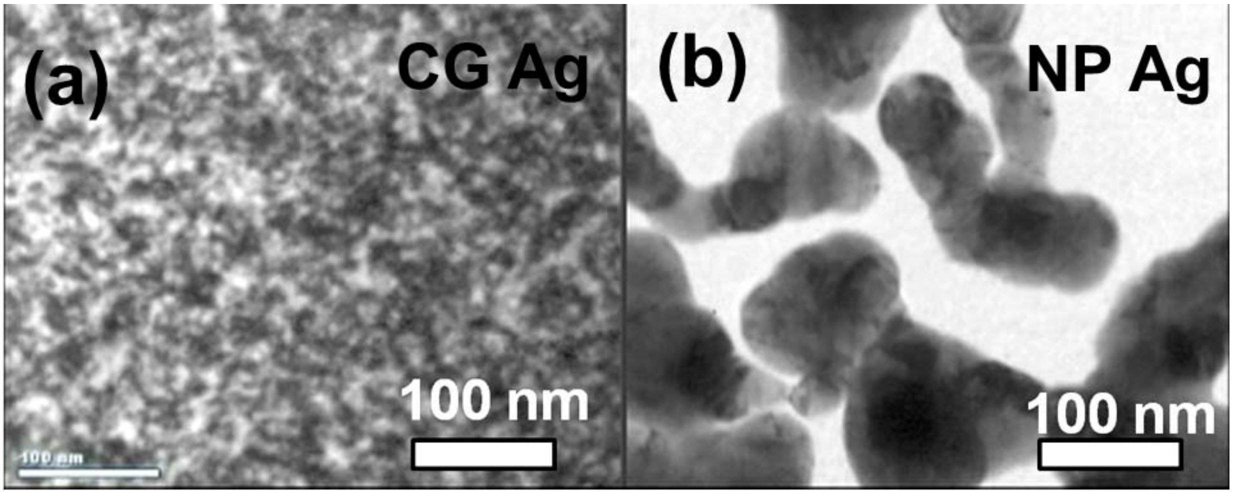

Supplementary Video 2. Comparison of microstructural evolution of CG and NP Ag during Kr ion irradiation at 0.020-0.038 dpa at energy of 1MeV. (a) and (b) show the microstructural evolution in CG and NP Ag under irradiation, respectively.

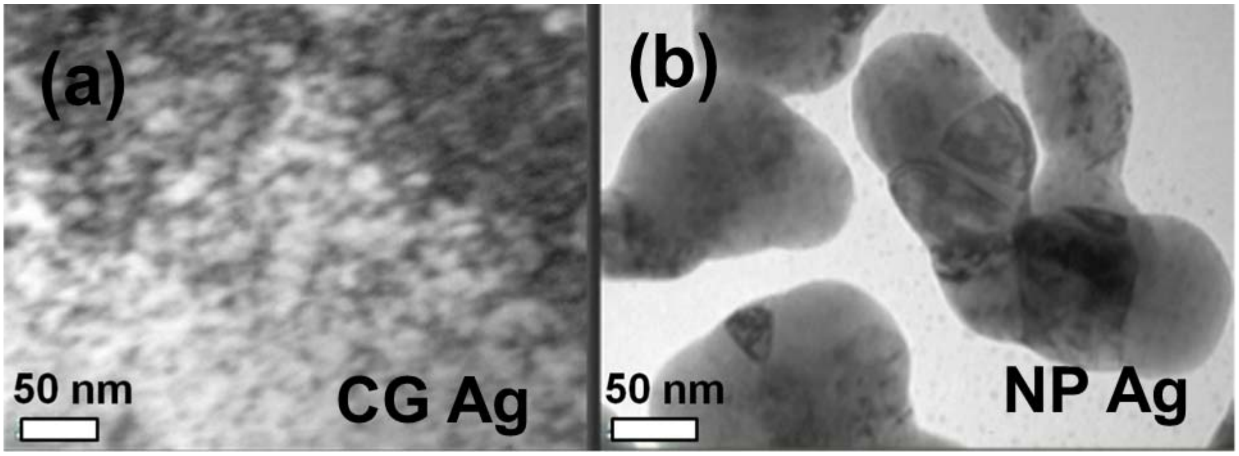

Supplementary Video 3. Microstructural evolution of CG (a) and NP (b) Ag during Kr ion irradiation at 1.000-1.024 dpa.

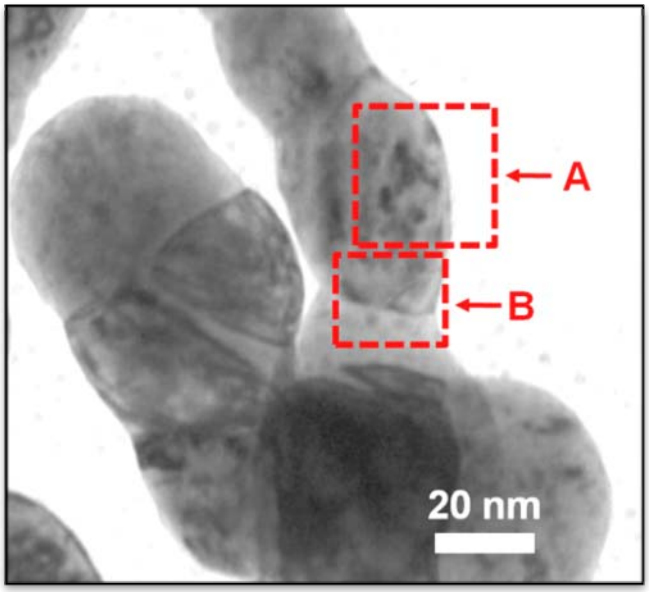

Supplementary Video 4. *In situ* video capturing the migration and removal of defect clusters in NP Ag irradiated to 1.17-1.30 dpa at energy of 1MeV. Region “A” shows the evidence of absorption of dislocation loop, stacking fault tetrahedral and dislocation segment by free surface. Region “B” shows the trapping of a dislocation loop by grain boundary and subsequent removal of the dislocation loop by a triple junction.

Calculation of image force and vacancy formation energy near free surface

We examine the interaction of discrete dislocation loops with free surface in an attempt to evaluate vacancy formation energy close to free surfaces. The image force of a dislocation loop adjacent to free surface provides driving force for its migration towards free surface[1]. Without losing general applicability, the image force per unit length (F/L) on an edge dislocation arising from free surface can be simplified as[2]:

$$\frac{F}{L} = -\frac{Gb^2}{4\pi(1-v)z}, \qquad (1),$$

where G is shear modulus, b and v are Burgers vector and Poisson's ratio, respectively, and z is the distance to free surface. The formation energy for a vacancy (W) in SAZ can be expressed as $W = \frac{F}{L} lb$, where l is assumed to be the magnitude of Burgers vector. z is ~ 4.5 nm as observed experimentally (Fig. 3a-c), the vacancy formation energy (W) is estimated to be 0.04 eV, which is much lower than that in bulk Ag, 0.99 eV [3].

Calculation of interaction energy between dislocation loops

Assuming there are two coplanar dislocation loops with outer edge separation distance L_{out}, the interaction energy per unit length (E_{inter}/L) between the loops can be described as[2]:

$$\frac{E_{inter}}{L} = \frac{Gb^2}{4\pi L}\{2[L_{out} - (L^2 + L_{out}{}^2)^{1/2}] + L\ln(\frac{L + (L^2 + L_{out}{}^2)^{1/2}}{-L + (L^2 + L_{out}{}^2)^{1/2}})\} \quad (2),$$

where the length L of dislocation is estimated as the diameter of the dislocation loop (d_{defect}). We can estimate the interaction energy (E_{inter}) as a function of separation distance of two defect clusters in irradiated CG and NP Ag (see supplementary Fig. S2). The average diameters of

defect clusters, 8 nm in CG Ag and 3 nm in NP Ag, are used in the calculation. Clearly E_{inter} decreases with increasing L_{out}. L_{out} can be estimated by using $L_{out} = L_s - d_{defect} = 1/\sqrt[3]{\rho} - d_{defect}$, where ρ is the defect cluster density (Fig. 2a) and L_s is the separation distance calculated by defect cluster density. By using the saturation defect cluster density and diameters, L_{out} is estimated to be 9 and 19 nm, respectively in CG and NP Ag. Correspondingly the interaction energy between defect clusters is estimated to be 0.85 eV in CG Ag, compared to 0.06 eV in NP Ag (supplementary Fig. S2).

Reference

1 Barnett, D. & Lothe, J. An image force theorem for dislocations in anisotropic bicrystals. *Journal of Physics F: Metal Physics* **4**, 1618 (1974).

2 Hirth, J. P. & Lothe, J. Theory of dislocations. *John Wiley and Sons, Inc., 1982*, 857 (1982).

3 Foiles, S., Baskes, M. & Daw, M. Embedded-atom-method functions for the fcc metals Cu, Ag, Au, Ni, Pd, Pt, and their alloys. *Physical Review B* **33**, 7983 (1986).
